# Acute Aerobic Swimming Exercise Induces Distinct Effects in the Contractile Reactivity of Rat Ileum to KCl and Carbachol

**DOI:** 10.3389/fphys.2016.00103

**Published:** 2016-03-18

**Authors:** Layanne C. da Cunha Araujo, Iara L. L. de Souza, Luiz H. C. Vasconcelos, Aline de Freitas Brito, Fernando R. Queiroga, Alexandre S. Silva, Patrícia M. da Silva, Fabiana de Andrade Cavalcante, Bagnólia A. da Silva

**Affiliations:** ^1^Programa de Pós-graduação em Biologia Celular e Molecular, Centro de Ciências Exatas e da Natureza, Universidade Federal da ParaíbaJoão Pessoa, Brazil; ^2^Programa de Pós-graduação em Produtos Naturais e Sintéticos Bioativos, Centro de Ciências da Saúde, Universidade Federal da ParaíbaJoão Pessoa, Brazil; ^3^Departamento de Educação Física, Centro de Ciências da Saúde, Universidade Federal da ParaíbaJoão Pessoa, Brazil; ^4^Departamento de Biologia Molecular, Centro de Ciências Exatas e da Natureza, Universidade Federal da ParaíbaJoão Pessoa, Brazil; ^5^Departamento de Fisiologia e Patologia, Centro de Ciência da Saúde, Universidade Federal da ParaíbaJoão Pessoa, Brazil; ^6^Departamento de Ciências Farmacêuticas, Centro de Ciências da Saúde, Universidade Federal da ParaíbaJoão Pessoa, Brazil

**Keywords:** acute exercise, contractile reactivity, gastrointestinal tract, rat ileum, swimming

## Abstract

Aerobic exercise promotes short-term physiological changes in the intestinal smooth muscle associated to the ischemia-reperfusion process; however, few studies have demonstrated its effect on the intestinal contractile function. Thus, this work describes our observations regarding the influence of acute aerobic swimming exercise in the contractile reactivity, oxidative stress, and morphology of rat ileum. Wistar rats were divided into sedentary (SED) and acutely exercised (EX-AC) groups. Animals were acclimated by 10, 10, and 30 min of swimming exercise in intercalated days 1 week before exercise. Then they were submitted to forced swimming for 1 h with a metal of 3% of their body weight attached to their body. Animals were euthanized immediately after the exercise section and the ileum was suspended in organ baths for monitoring isotonic contractions. The analysis of lipid peroxidation was performed in order to determinate the malondialdehyde (MDA) levels as a marker of oxidative stress, and intestinal smooth muscle morphology by histological staining. Cumulative concentration-response curves to KCl were altered in the EX-AC with an increase in both its efficacy and potency (*E*_max_ = 153.2 ± 2.8%, *EC*_50_ = 1.3 ± 0.1 × 10^−2^ M) compared to the SED group (*E*_max_ = 100%, *EC*_50_ = 1.8 ± 0.1 × 10^−2^ M). Interestingly, carbachol had its efficacy and potency reduced in the EX-AC (*E*_max_ = 67.1 ± 1.4%, *EC*_50_ = 9.8 ± 1.4 × 10^−7^ M) compared to the SED group (*E*_max_ = 100%, *EC*_50_ = 2.0 ± 0.2 × 10^−7^ M). The exercise did not alter the MDA levels in the ileum (5.4 ± 0.6 μ mol/mL) in the EX-AC compared to the SED group (8.4 ± 1.7 μ mol/mL). Moreover, neither the circular nor the longitudinal smooth muscle layers thickness were modified by the exercise (66.2 ± 6.0 and 40.2 ± 2.6 μm, respectively), compared to the SED group (61.6 ± 6.4 and 34.8 ± 3.7 μm, respectively). Therefore, the ileum sensitivity to contractile agents is differentially altered by the acute aerobic swimming exercise, without affecting the oxidative stress and the morphology of ileum smooth muscle.

## Introduction

In recent decades, human behavioral changes has promoted the development of inappropriate habits such as sedentary lifestyle, which increasingly affects the quality of life. Thus, the exercise has become a growing practice and considered as an important therapeutic approach in the prevention and treatment of different diseases, such as gastrointestinal diseases, diabetes, obesity, cardiovascular, and respiratory disorders (Sessa et al., 1994; Casaburi et al., 1997; Booth et al., 2000; Howley, 2001; Peters and Vries, 2001; Seo et al., 2014).

Continuous aerobic exercise stimulates adaptations in the cardiovascular, respiratory, and muscular systems in order to maintain the tissue oxygen demand, related to structural and functional changes, categorizing as chronic exercise (Hermansen and Wachtlova, 1971; Brodal et al., 1977; Howley, 2001). Conversely, isolated sessions of aerobic exercise, which promote health-related changes hours after the exercise, are classified as acute exercise (Howley, 2001).

The aerobic exercise can subject several organs to a process of ischemia-reperfusion due to the diversion of blood flow to the skin and the active skeletal muscle. Accordingly, although the intestinal smooth muscle is not directly involved in physical exercises, it is submitted to this physiological stress related to the process of ischemia-reperfusion (Otte et al., 2001). Hence, it is described that the exercise can promote ischemia and motor abnormalities in both gut and intestinal mucosa (Ballabeni et al., 2002).

Physiological stress promoted by acute exercise has shown to promote oxidative stress, while in chronic exercise there is an increase of antioxidant defenses, which are able to remove these free radicals and stabilize the production of reactive species (Fisher-Wellman and Bloomer, 2009). Those free radicals produced react with polyunsaturated fatty acids in cell membranes, process known as lipid peroxidation, which modifies the permeability, fluidity, and integrity of membrane, affecting then the cell functionality (Mahattanatawee et al., 2006).

Regarding the effects of aerobic exercise on gastrointestinal tract, some studies have shown this influence on intestinal contractile responsiveness in a chronic approach (Rosa et al., 2005; de Lira et al., 2008; Araujo et al., 2015). On the other hand, studies focus on the acute effects of exercise on absorption of nutrients, intestinal mucosa, and gastrointestinal permeability (Pals et al., 1997; Lambert et al., 2001). Various changes in gastrointestinal motility are reported to occur during exercise, as increasing gastric emptying in high-intensity exercise (Leiper et al., 2001). Additionally, study with humans demonstrated that the intestinal motility increases after acute exercise (Rao et al., 1999). Other studies have related the occurrence of gastrointestinal symptoms, such as abdominal cramps, diarrhea in corridors, and constipation, in sedentary people (Riddoch and Trinick, 1998). These observations suggest that exercise, especially acute aerobic exercise, stimulates intestinal motor activity, and facilitate defecation; however, there are few evidences to confirm them and the mechanisms related to these symptoms are still unknown.

The contractile reactivity of intestinal smooth muscle is stimulated by electrical activity promoted by slow waves that represent a continuous change in the membrane potential. These waves are generated by the interstitial cells of Cajal (ICC), which promote a change in potential from −80 to −40 mV (Cheng et al., 2010). The KCl is a salt present in the extracellular medium and stimulates the membrane depolarization, promoting the opening of voltage-dependent Ca^2+^ channels and then the calcium influx leading to intestinal smooth muscle contraction (Rembold, 1996). In addition to the electrical stimulation, smooth muscle contraction occurs by a pharmacomechanical coupling stimulated by acetylcholine (ACh) released from myenteric plexus, or by stimulation with its analog, carbachol (CCh), which binds to M_3_ receptor, leading to production of secondary messengers, which operates in the development of contraction force (Somlyo and Somlyo, 1994).

Therefore, this study aimed to investigate the influence of acute aerobic swimming exercise on the contractile reactivity of rat intestinal smooth muscle for electro- and pharmacomechanical couplings, KCl, and CCh, respectively, by functional, biochemical and morphological approaches.

## Materials and methods

### Animals

Wistar rats (*Rattus norvegicus*), 3 months old, weighing 200–250 g, were obtained from the Instituto de Pesquisa em Fármacos e Medicamentos (IPeFarM/UFPB) animal facilities. The animals were kept under a food control with a balanced diet (Labina®) and had access to water *ad libitum*. They were maintained on controlled ventilation and temperature (21 ± 1°C) and were subjected to a daily light-dark cycle of 12 h environment. After the last exercise session, animals were euthanized by cervical dislocation followed by cervical vessels section, then the ileum was removed and segmented. All experimental procedures were performed following the principles of animal care of the “Guidelines for the ethical use of animals in applied etiology studies” (Sherwin et al., 2003) and previously approved by the Ethics Committee on Animal Use (CEUA/UFPB n° 0907/13).

### Exercise protocol

The animals were divided into sedentary (SED) and submitted to acute exercise (EX-AC) groups (*n* = 5). They were acclimated 1 week before exercise, being subjected to periods of 10, 10, and 30 min of swimming exercise, three times a week during 1 week, in intercalated days, according to the protocol adapted from Chibalin et al (2000). The swimming protocol was adapted from Chies et al (2003) and Brito et al (2015). Briefly, the animals were submitted to forced swimming for 1 h with a metal of 3% of their body weight attached to them, to improve the animal's resistance and avoid floating in water, however maintaining the aerobic exercise as moderate intensity (Brito et al, 2015). The exercise was performed in a plastic container measuring 43 cm width, 63 cm length, and 33 cm depth, with water at 24–27°C. SED (control) group was subjected to the same stress as the exercised animals, including food deprivation, exposure to noise, and were placed in a container with 1.5 cm of water at 24–27°C for 2 min, in order to mimic the contact of the animal with water. Animals were euthanized immediately after the exercise (EX-AC) or stress (SED) period and ileum was removed (Gobatto et al., 2001; Lima et al., 2013).

### Contractile reactivity measurement

Ileum segments (2–3 cm) were individually suspended in organ bath containing Tyrode solution gassed with a carbogen mixture (95% O_2_ and 5% CO_2_) at 37°C, kept under 1 g resting tension for 30 min (Radenkovic et al., 2006). The Tyrode solution composition (in mM) was: NaCl (150.0), KCl (2.7), CaCl_2_.2H_2_O (1.8), MgCl_2_.6H_2_O (2.0), NaHCO_3_ (12.0), NaH_2_PO_4_ (0.4), D-glucose (5.5). To register the isotonic contractions, organs were suspended by cotton yarn in organ baths and recorded on smoked drum through levers coupled to kymographs (DTF, Brazil). Baths were attached to a thermostatic pump Polystat 12002 Cole-Palmer (Vernon Hills, IL, USA) for temperature control.

After the organ stabilization period, an isotonic contraction was induced with 30 mM KCl to verify the functionality of the organ. The contractile reactivity was assessed based on *E*_max_ (maximum effect) and *EC*_50_-values (concentration of a substance that produces 50% of its maximal effect) calculated from two similar cumulative concentration-response curves to KCl (electromechanical coupling) or CCh (pharmacomechanical coupling). The maximum amplitude of the cumulative curves was considered as 100% (control), and the EX-AC was assessed referring to it.

### Lipid peroxidation assay

Thiobarbituric acid reactive substances (TBARS) are formed as product of lipid peroxidation (isoprostanes, lipid hydroperoxides, aldehydes, oxidized phospholipids), which can be detected by the TBARS assay using thiobarbituric acid as a reagent. Lipid peroxidation in tissue was determined measuring the chromogenic product of the 2-thiobarbituric acid (TBA) reaction with malondialdehyde (MDA; Winterbourn et al., 1985; Chang et al., 1997). Ileum segments were homogenized with KCl (1:1) and a sample (250 μL) was incubated at 37°C for 60 min. Then, the mixture was precipitated with 35% perchloric acid and centrifuged at 0.02 G for 20 min at 4°C. The supernatant was collected and 0.6% TBA (400 μL) were added and incubated at 95–100°C for 1 h. After cooling, the samples were read in spectrophotometer (532 nm). The results were expressed in μ mol/L of dry tissue.

### Histological analysis

Samples of ileum were fixed in 10% formaldehyde solution, subjected to standard histological procedures as follow: (1) dehydration, by increasing alcohol series of 70% for 24 h, 80, 96, and 100% (third bath) for 1 h each; (2) diaphanization, by bath in 100% xylene alcohol (1:1) for 1 h, followed by two baths in pure xylene for 1 h each; and (3) embedding in paraffin by passing the tissue through two baths of liquid paraffin (heated to 50°C) for 1 h each. Then, the tissues were embedded in a new paraffin. The blocks obtained were cut to 5 μm thick in cross section of the ileum and the sections were stained with Mayer's hematoxylin/eosin. The second quadrant of the ileum circumference was analyzed and measurements of the circular and longitudinal muscle layers were obtained in an image analysis program.

### Statistical analysis

Data were expressed as the mean and standard error of the mean (SEM.). *EC*_50_ and *E*_max_ were calculated by nonlinear regression (Neubig et al., 2003). Analysis were performed using the unpaired Student's *t*-test, and the null hypothesis was rejected when *p* < 0.05. Data were analyzed by GraphPad Prism® version 6.0 software and the visualization of histological sections was performed on Q-Capture® Pro version 7.0 software.

## Results

### Ileum contractile reactivity

**Figure 1:**
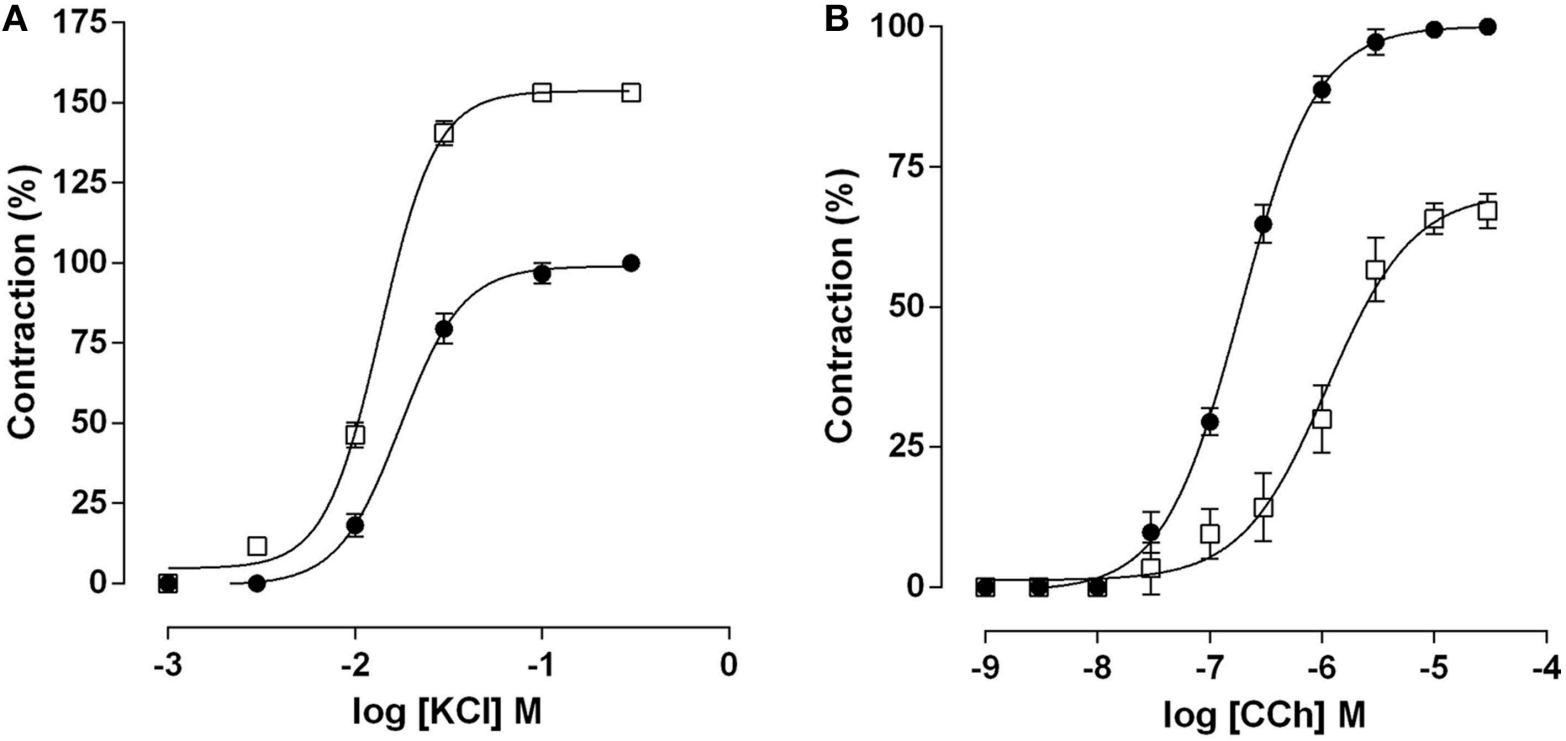
**Cumulative concentration-response curves to KCl (A) and CCh (B) in both SED (●) and EX-AC groups (□) in rat ileum**. The symbols and vertical bars represent the mean and *SD*, respectively, (*n* = 5).

Cumulative concentration-response curve to KCl (10^−3^–10^−1^ M) was leftward shifted in the EX-AC group compared with the control (Figure 1A), with *E*_max_ increase and *EC*_50_ reduction (Table 1). Conversely, the cumulative concentration response curve to CCh (10^−9^–10^−4^ M) was rightward shifted in the EX-AC group compared to the control (Figure 1B), with *E*_max_ reduction and *EC*_50_ increase (Table 1).

**Table 1:** **Values of *EC*_50_ (M) and *E*_max_ (%) of KCl and CCh in both SED and EX-AC groups in rat ileum**.

	**KCl**		**CCh**	
	***EC*_50_ (M)**	***E*_*max*_ (%)**	***EC*_50_ (M)**	***E*_*max*_ (%)**
SED	1.8 ± 0.1 × 10^−2^	100.0	2.0 ± 0.2 x 10^−7^	100.0
EX-AC	1.3 ± 0.1 × 10^−2^***^^	153.2 ± 2.8^***^	9.8 ± 1.4 x 10^−7^***^^	67.1 ± 1.4^***^

Student's t-test: EC_50_ for KCl

*** p < 0001 (SED vs. EX-AC);

for CCh:

*** p < 0.001 (SED vs. EX-AC);

E_max_ for KCl

*** p < 0,001 (SED vs. EX-AC);

E_max_ for CCh

*** *p < 0.0001 (SED vs. EX-AC), (n = 5)*.

### Lipid peroxidation assay

The MDA levels in rat ileum had no significant difference between the SED (8.4 ± 1.7 μ mol/L) and EX-AC (5.4 ± 0.6 μ mol/L**)** groups.

### Histological analysis

**Figure 2:**
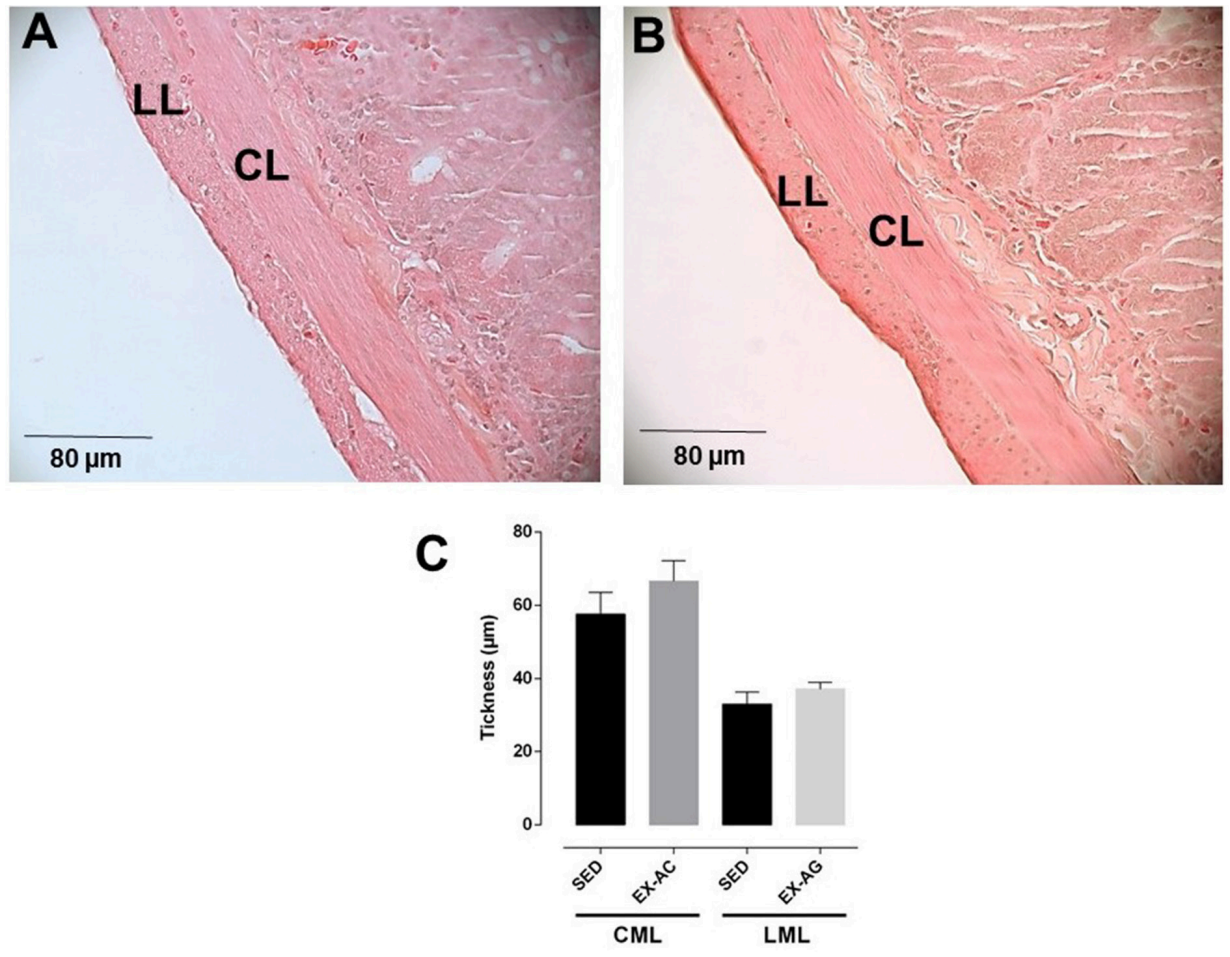
**Histological section of rat ileum from SED (A) and EX-AC (B) groups and thickness of circular muscle layer (CML) and longitudinal muscle layer (LML) (C) of the rat ileum**. Increased lens, 40x; LML, longitudinal muscle layer; CMC, circular muscle layer. The columns and vertical bars represent the mean and *SD*, respectively, (*n* = 5).

The thickness of the circular smooth muscle layer of rat ileum had no significant difference between the SED (57.6 ± 6.0 μm) and EX-AC groups (66.6 ± 5.6 μm). Similarly, the longitudinal smooth muscle layer showed no significant difference between the SED (33.0 ± 3.4 μm) and EX-AC groups (37.1 ± 1.7 μm; Figure 2).

## Discussion

In this study, we investigated the influence of acute aerobic swimming exercise on the contractile reactivity, oxidative stress, and morphology of rat ileum, and we showed that this modality of exercise produces different changes in the rat ileum reactivity to electro- and pharmacomechanical couplings without altering lipid peroxidation and organ morphology.

Swimming is an useful exercise modality to identify some physiological, biochemical, and molecular alterations caused by the physical activity, especially the chronic training (Baar et al., 2002; Iemitsu et al., 2002; Jones et al., 2003). Besides that, experiments with human have been important to evaluate these changes (Gobatto et al., 2001, 2008; Voltarelli et al., 2002; Araujo et al., 2007). It is known that individuals who practice swimming can present gastrointestinal alterations (Pyne et al., 2014), however, the precise mechanism involved in these effects remains unclear and there is a lack of studies showing the effects of the acute exercise in this system.

Physical exercise promotes gastrointestinal complications, such as diarrhea, caused by increased gastrointestinal motility; moreover, the smooth muscle is the responsible for the intestinal motor activity. Thus, we launched the hypothesis that the acute swimming exercise may promote changes in contractile reactivity of rat ileum. Our results showed that while the exercise increased the contractile reactivity of rat ileum to KCl, a contractile agent that acts by an electromechanical mechanism, by increasing both the efficacy and relative potency, it impaired the contractile response to CCh, a pharmacomechanical contractile agent, evidenced by the decreased efficacy, and relative potency. The question is how these differential responses of the ileum smooth muscle can explain the gastrointestinal episodes during the exercise.

The intestinal smooth muscle cell responds to the stimulation promoted by the myenteric plexus, which releases ACh to contract this tissue. In addition, pacemaker of interstitial cells of Cajal located at the boundaries and in the substance of the inner circular muscle layer, from which they spread to the outer longitudinal muscle layer, are responsible for the slow waves that cause intestinal movement (Murthy, 2006). Thus, we assume that the dual effect of acute swimming exercise on the rat ileum could be attributed to a raised stimulation of the pacemaker cells during the exercise, which makes the resting membrane potential less negative and the cells more responsible for the electromechanic coupling. Furthermore, it is reasonable to say that the ACh release from myenteric plexus may be increased during the exercise, which promotes a desensitization of muscarinic receptors in the smooth muscle, leaving the cells less responsive to the exogenous cholinergic stimulus (e.g., carbachol). However, these are preliminary data and require further experiments to confirm or exclude this hypothesis. Ballabeni et al. (2002) also described different responses to KCl or CCh stimuli in a distinct protocol, in which rats were subjected to 1 h occlusion of superior mesenteric artery plus interruption of collateral blood flow and reperfusion that presented suppression on ACh-induced contractile response after 24 h, however, not on the KCl-induced contraction. Thus, it indicates a possible role of the reperfusion process as the main responsible for the altered contractile response to muscarinic agonist.

Aerobic exercise can lead to free radicals increased production, reactive oxygen (ROS) and nitrogen (NRS) species, causing oxidative stress with potential damage to various organs (Liu et al., 2000). The ischemia reperfusion process along the exercise can cause changes in several organs, such as lungs, kidneys, liver, and intestine (Kiliç et al., 2015). Thus, launched the hypothesis that acute swimming exercise promotes oxidative stress and leads to changes in the rat ileum morphology; however, we observed that the exercise did not imbalance the reactive oxygen species in the ileum. Additionally, the exercise did not alter the structure of the intestinal smooth muscle tissue, confirmed by the histological analysis, i.e., the fast time of exercise in a single session is not able to promote stress and alter the morphology of rat small intestine.

In summary, the acute exercise, swimming mode, promotes a dual effect on the responsiveness of rat ileum to electro- and pharmacomechanical couplings, which can modify the intestinal motility. Furthermore, although the exercise can promote oxidative stress and morphological changes in some organs due to ischemia-reperfusion process, that dual effect was not accompanied by neither an oxidative stress increase nor morphological changes of rat ileum. Furthermore, other contractile agonists may be employed for better describing the influence of this modality of exercise on gastrointestinal tract. This approach will provide information about mechanisms involved in this effect.

## Author contributions

LA: developed experiments and wrote the manuscript. IS: developed experiments. LV: developed experiments. AB: developed experiments. FQ: developed experiments. AS: collaborating professor of part of lipid peroxidation. PS: collaborating professor of histology part. FC: participated of writing of manuscript. BS: developed the preparation of work and participated of writing the manuscript.

### Conflict of interest statement

The authors declare that the research was conducted in the absence of any commercial or financial relationships that could be construed as a potential conflict of interest.
